# Inhibitory effect and safety of *Angelica dahurica–Ligusticum sinense* extracts against *Malassezia furfur* and its biofilms

**DOI:** 10.1371/journal.pone.0357601

**Published:** 2026-09-15

**Authors:** Jinyan Zhang, Yingzheng Wang, Yinghao Wang, Borui Chen, Huan Li

**Affiliations:** 1 Dermatology Department, Fujian Provincial Geriatric Hospital, Fuzhou, Fujian, China; 2 College of Pharmacology, Fujian University of Traditional Chinese Medicine, Fuzhou, Fujian, China; 3 Dermatology Department, Dermatology Hospital of Fuzhou, Fuzhou First General Hospital Affiliated with Fujian Medical University, Fuzhou, Fujian, China; 4 Department of Infectious, Fujian Provincial Geriatric Hospital, Fuzhou, Fujian, China; Yenepoya University, INDIA

## Abstract

**Objective:**

To evaluate the in vitro inhibitory activity of different *Angelica dahurica–Ligusticum sinense* extracts (ethanolic, aqueous, and cold-pressed) against planktonic cells and biofilms of *Malassezia furfur*.

**Methods:**

Ultra-performance liquid chromatography (UPLC) was used to quantify the major constituents. Micro-broth dilution determined the minimum inhibitory concentration (MIC), and time–kill assays assessed fungicidal kinetics. Crystal violet staining assay (CVA) and XTT reduction assays quantified inhibition of biofilm biomass formation/metabolic activity and reduction of mature biofilms. Dermal irritation of the ethanolic extract was evaluated in rats.

**Results:**

UPLC showed that the ethanolic extract contained the highest levels of ferulic acid, bergapten, oxypeucedanin, imperatorin, and isoimperatorin. The MIC of the ethanolic extract was 5 mg/mL, superior to those of the aqueous and cold-pressed extracts (10 mg/mL each). A time–kill study revealed rapid fungicidal activity at 8 × MIC. Biofilm assays demonstrated that 0.5× and 1 × MIC of the ethanolic extract significantly inhibited biofilm formation (P < 0.05), whereas 8 × MIC effectively eradicated pre-formed biofilms, markedly reducing biomass and metabolic activity (P < 0.01). No cutaneous irritation was observed in rats at 8 × MIC.

**Conclusion:**

The *A. dahurica-L. sinense ethanolic* extract potently inhibited and eradicated *M. furfur* planktonic cells and biofilms with favorable safety. This activity may be attributable to its high content of coumarins and phenolic acids, suggesting its potential as a natural agent for the treatment of *M. furfur*-associated skin disorders.

## 1. Introduction

*Malassezia furfur* is a lipophilic, yeast-like fungus that constitutes a ubiquitous component of the human skin microbiome, with a particular predilection for sebum-rich regions such as the scalp, face, and chest [1]. This organism has been implicated in a spectrum of dermatoses, including dandruff, seborrheic dermatitis, and pityriasis versicolor, which impair quality of life and frequently develop into chronic or relapsing inflammatory conditions [2]. Epidemiological data indicate that approximately 50% of adults worldwide experience dandruff, whereas the prevalence of seborrhoeic dermatitis in the general population ranges from 1% to 3% [3].

Pathogenically, *M. furfur* secretes lipases that hydrolyse sebum triglycerides into free fatty acids, thereby triggering cutaneous inflammation [4]. Equally important, its ability to form robust biofilms markedly enhances resistance to both host immune effectors and conventional antifungal agents, constituting a critical driver of therapeutic failure and disease recurrence [5,6].

Current standard-of-care relies primarily on topical azoles (e.g., ketoconazole) and selenium sulfide. Although these agents confer moderate efficacy, prolonged use is associated with emerging drug resistance, irritant reactions, and perturbation of the cutaneous micro-ecology [7]. Moreover, biofilm-mediated impermeability severely limits drug penetration, an obstacle that contributes into clinical failure rates of 60–80% for biofilm-associated infections [8,9]. Consequently, the identification of natural alternatives capable of inhibiting *M. furfur* growth and disrupting its biofilms has become a priority in dermatological research [10].

Traditional Chinese medicine (TCM) has a long-standing history and unique strengths in the management of dermatoses. Its multi-component, multi-target profile provide a promising strategy for overcoming antimicrobial resistance and biofilm-related recalcitrance [11]. *Angelica dahurica* (Bai-Zhi) and *Ligusticum sinense* (Gao-Ben), two classical TCM agents, are frequently prescribed for cutaneous inflammation and infectious disorders [12]. Ming-dynasty *Bian Min Tu Zuan* records: “For dry dandruff: mix equal parts of Gao-Ben and Bai-Zhi, powder them, scatter into the hair at night, comb the next morning, the dirt will be removed.” A similar formula was reiterated in the Qing-dynasty *De Pei Materia Medica*: “Gao-Ben combined with Bai-Zhi powder, rubbed at night and combed at dawn, eliminates greasy scales and white flakes.” Thus, the combination of *Angelica dahurica* and *Ligusticum sinense* has a long history in treating dandruff. However, the traditional method of grinding the herbs into powder is cumbersome and inconvenient. Moreover, its inhibitory effect on *M. furfur* and the underlying mechanism remain unclear [13].

To address these gaps, we prepared Angelica dahurica and Ligusticum sinense extracts by three different protocols (ethanolic, aqueous, and cold-pressed) and evaluated their antifungal activity in vitro, minimum inhibitory concentration (MIC), and capacity both to inhibit and to disrupt *M. furfur* biofilms. Scanning electron microscopy (SEM) and crystal-violet staining were employed to visualize structural alterations in biofilm architecture. The findings will provide experimental evidence for the development of natural antifungal agents and lay a foundation for TCM-based formulations aimed at modulating the skin micro-ecosystem.

## 2. Materials and methods

### 2.1. Preparation of extracts

#### 2.1.1. Preparation of ethanolic and aqueous extracts.

Coarsely powdered *Angelicae dahuricae* Radix and *Ligustici sinensis* Rhizoma et Radix (1:1, w/w) were accurately weighed (25g total) and macerated in 3 volumes (v/w) of either 80% ethanol or distilled water for 12h at room temperature. After maceration, the mixtures were reflux-extracted at 85°C for 2 h*,* the procedure was repeated three times. The combined filtrates were concentrated under reduced pressure and lyophilized. The resulting powders were stored at 4 °C until use. Yield of dry extract: 3.0756 g crude drug per g freeze-dried ethanolic extract, and 4.1586 g crude drug per g freeze-dried aqueous extract.

#### 2.1.2. Preparation of cold-percolated extract.

Coarsely powdered drug (25 g) was moistened with 1 volume of 80% ethanol for 2 h, packed into a percolator, and allowed to macerate with 8 volumes of 80% ethanol for 12 h. Percolation was then carried out at a flow rate of 3 mL/min. The percolate was pooled, concentrated in vacuo, and lyophilized. Yield: 3.035 g crude drug per g freeze-dried cold-percolated extract.

### 2.2. Chemical characterization of extracts

#### 2.2.1. Screening and optimization of chromatographic conditions.

Mobile-phase systems were systematically evaluated. Acetonitrile–0.1% formic acid in water was selected because it provided symmetrical peak shape, stable baseline, and optimal resolution for the five marker compounds: ferulic acid, bergapten, oxypeucedanin, imperatorin, and isoimperatorin.

#### 2.2.2. Final UPLC conditions.

An ACQUITY UPLC BEH C18 column (2.1 × 100 mm, 1.7 µm) was used at 35°C. The mobile phase consisted of acetonitrile and 0.1% aqueous formic acid, delivered at 0.25 mL min^−1^. UV detection was performed at 302 nm, injection volume was 10 µL.

#### 2.2.3. Preparation of reference standard solutions.

Accurately weighed amounts of reference standards 0.57 mg of ferulic acid, 0.47 mg of bergapten, 1.16 mg of imperatorin, 3.55 mg of oxypeucedanin, and 0.78 mg of isoimperatorin—were placed into a 10 mL volumetric flask. Methanol was added to prepare a stock solution with final concentrations of 0.057, 0.047, 0.116, 0.355, and 0.078 mg/mL for each compound, respectively. Then, 1 mL, 2 mL, 5 mL, 1 mL, and 1 mL of these five individual stock solutions were precisely transferred into another 10 mL volumetric flask, mixed well, to obtain the mixed reference standard solution, which was stored at 4°C.

#### 2.2.4. Preparation of test sample solutions.

Accurately weighed amounts of the freeze-dried powders of the ethanol extract, water extract, and percolation extract—21.56 mg, 13.39 mg, and 17.70 mg, respectively—were dissolved in 50% methanol to prepare sample solutions with concentrations of 0.5390 mg/mL, 0.6695 mg/mL, and 0.5531 mg/mL, respectively. The solutions were filtered through a 0.22 μm microporous membrane to obtain the final test sample solutions.

#### 2.2.5. Linearity investigation.

The mixed reference standard solution was precisely aspirated and sequentially diluted 2, 4, 8, 16, 32, and 64 times with 50% methanol. Each dilution was injected for analysis, and the peak areas were recorded. Standard curves were plotted with the concentration of the reference standard solution as the x-axis and the peak area as the y-axis. Regression equations were calculated.

#### 2.2.6. Content determination in samples.

The three test sample solutions were injected for analysis. The peak areas obtained from the chromatograms were used to calculate the mass fractions of the five components in each sample based on the linear regression equations.

### 2.3. Fungal strain

The Malassezia strains used in this study were obtained from the archived culture collection of the Dermatology Prevention and Treatment Hospital, Fuzhou First General Hospital (MF-FZ12). These isolates were originally recovered from patients with tinea versicolor during a previous study, for which ethical approval had been obtained. The current in vitro experiments were conducted solely on stored isolates, and no additional human subjects or clinical data were involved.Species identification was confirmed by PCR amplification and sequencing of the ribosomal DNA internal transcribed spacer (ITS).

### 2.4. Antimicrobial and anti-biofilm activity of extracts

#### 2.4.1. Determination of minimal inhibitory concentration (MIC).

MICs were measured by a micro-broth dilution method. Briefly, two-fold serial dilutions of each extract were prepared in Leeming–Notman broth in 96-well plates. An equal volume of 1–5 × 10^5^CFU/mL *M. furfur* suspension was added. After 48 h at 32 °C, growth was assessed visually, and the lowest concentration showing no turbidity was recorded as the MIC.

#### 2.4.2. Time–kill kinetics.

Time–kill assays were performed by withdrawing aliquots at 2, 4, 8, 12, and 24 h and quantifying viable *M. furfur* by plate counts (colony-forming units, CFU) to evaluate fungistatic/fungicidal kinetics of the extract at 1 × , 4 × , and 8 × the minimum inhibitory concentration (MIC).

#### 2.4.3. Crystal violet staining assay (CVA) for inhibition of biofilm biomass.

A single colony was inoculated into 10 mL mDixon broth in a 15 mL conical tube and adjusted to an OD600 of 0.5. Aliquots of 100 µL cell suspension were dispensed into 96-well plates, followed by 100 µL of the test extract at the indicated concentrations. Plates were incubated at 37 °C for 48 h. Biofilms were fixed with methanol for 5 min, the fixative was removed, and wells were rinsed twice with physiological saline. Crystal violet solution was then added and incubated for 30 min. After removing the stain and rinsing twice with physiological saline to eliminate residual dye, bound crystal violet was solubilized with glacial acetic acid at room temperature for 5 min, and absorbance was recorded at 570 nm.

#### 2.4.4. XTT reduction assay for inhibition of biofilm metabolic activity.

A single colony was inoculated into 10 mL mDixon broth in a 15 mL conical tube and adjusted to an OD600 of 0.5. Aliquots of 100 µL cell suspension were added to 96-well plates and mixed with 100 µL of the extract at the indicated concentrations. Plates were incubated at 37°C for 48 h. After incubation, the XTT assay was performed according to the manufacturer’s instructions (XTT kit, KeyGEN Biotech). Briefly, 20 µL XTT reagent was added per 100 µL medium in each well, incubated in the dark at room temperature for 30 min, and the absorbance was measured at 450 nm.

#### 2.4.5. Microscopic evaluation of biofilm inhibition.

A single colony was inoculated into 10 mL mDixon broth in a 15 mL conical tube and adjusted to an OD600 of 0.5. In 24-well plates, 200 µL of the cell suspension was combined with the corresponding concentrations of the extract and incubated at 37°C for 48 h. Biofilms were stained with crystal violet for 30 min, gently rinsed with physiological saline, and examined under a light microscope to assess cellular adhesion.

#### 2.4.6. Activity against preformed biofilms.

To establish preformed biofilms, M. furfur cell suspensions were dispensed into 96-well plates and incubated at 37°C for 48 h without the extracts. The culture medium and non-adherent cells were then removed, and the wells were washed twice with physiological saline. Fresh medium containing the extracts was added, and the preformed biofilms were treated at 37°C. After treatment, residual biofilm biomass and metabolic activity were quantified using the CVA and XTT reduction assay, respectively. For microscopic examination, preformed biofilms established in 24-well plates were treated under the same conditions, stained with crystal violet, and examined using a light microscope. Untreated preformed biofilms served as the negative control.

### 2.5. Dermal toxicity evaluation of ethanolic extract in rats

Eight SD rats (equal sexes) were randomly assigned to either the test group (8 × MIC ethanolic extract) or the vehicle-control group. Hair was clipped from both flanks (4 cm × 7 cm). Test sites received 0.5 mL of extract or solvent, were occluded with non-irritating dressings for 4 h, and then washed. Erythema and oedema were scored at 1, 24, 48 and 72 h post-removal using the Draize scale. The primary irritation index (PII) was calculated as: PII = (sum of all erythema scores + sum of all oedema scores)/total number of animals.

Scoring criteria
  Erythema: 0 = none, 1 = slight, 2 = moderate, 3 = severe, 4 = dark-red eschar.
  Oedema: 0 = none, 1 = slight, 2 = moderate, 3 = severe, 4 = bullous.
Interpretation
  PII 0–0.49 = non-irritant*,* 0.5–2.99 = mild irritant*,* 3.0–5.99 = moderate irritant.

### 2.6. Statistical analysis

All experiments were performed with at least three independent biological replicates, and each condition was tested in technical triplicate unless otherwise stated. Statistical data are presented as the mean ± SEM and were analyzed by one-way ANOVA with post hoc Bonferroni corrections for multiple comparisons using SPSS 11.0. A p-value of less than 0.05 was considered statistically significant.

## 3. Results

### 3.1. Quantification of the five marker compounds in the different extracts

The concentrations of the five bioactive constituents are summarized in Table 1. The ethanolic extract exhibited the highest levels of all analytes, with isoimperatorin being the most abundant (25.81 ± 0.12 mg/g dry extract), followed by imperatorin and ferulic acid. In contrast, only trace ferulic acid (0.42 ± 0.03 mg/g) was detected in the aqueous extract, whereas the remaining four coumarins were below the limit of quantification. The percolated extract displayed intermediate values for all compounds, confirming that ethanol-rich solvents are superior for recovering the lipophilic coumarins responsible for the observed antifungal activity.

**Table 1:** Content of five chemical constituents in *Angelica dahurica–Ligusticum sinense* extracts (mg·g^−1^, mean values).

Extraction method	Mass fraction/(mg·g^−1^)				
	Ferulic acid	Bergapten	Oxypeucedanin	Imperatorin	Isoimperatorin
Ethanolic	4.1420	0.5748	0.5847	7.9873	25.8118
Aqueous	0.4156	/	/	/	/
Percolation	2.3907	0.2034	0.4719	6.4184	17.4208

### 3.2. Antimicrobial activity of different extracts against *M. furfur*

#### 3.2.1. Minimum inhibitory concentration (MIC).

All three extracts exhibited inhibitory effects against *M. furfur*(Table 2). The MIC of the *A. dahurica-L. sinense* ethanolic extract was 5 mg/mL, whereas the MICs of both the aqueous and percolated extracts were 10 mg/mL. These results indicate that the ethanolic extract had stronger antimicrobial activity than the aqueous and percolated extracts.

**Table 2:** MIC of *Angelica dahurica–Ligusticum sinense* Extracts against *Malassezia.*

Extracts	Drug concentration (mg/ml)												MIC (mg/ml)
	40	20	10	5	2.5	1.25	0.63	0.31	0.16	0.08	M.F	mDix	
AEE	0	0	0	0	1	2	3	4	4	4	4	0	5
AAE	0	0	0	1	1	2	3	4	4	4	4	0	10
APE	0	0	0	1	1	2	3	4	4	4	4	0	10

Note: 0 optically clear, 1 slightly turbid, 2 turbidity significantly reduced (50%), 3 turbidity slightly reduced, 4 no reduction in turbidity.

AEE: ethanolic extract, AAE: aqueous extract, APE: percolation extract.

#### 3.2.2. Time-kill curves.

**Fig 1:**
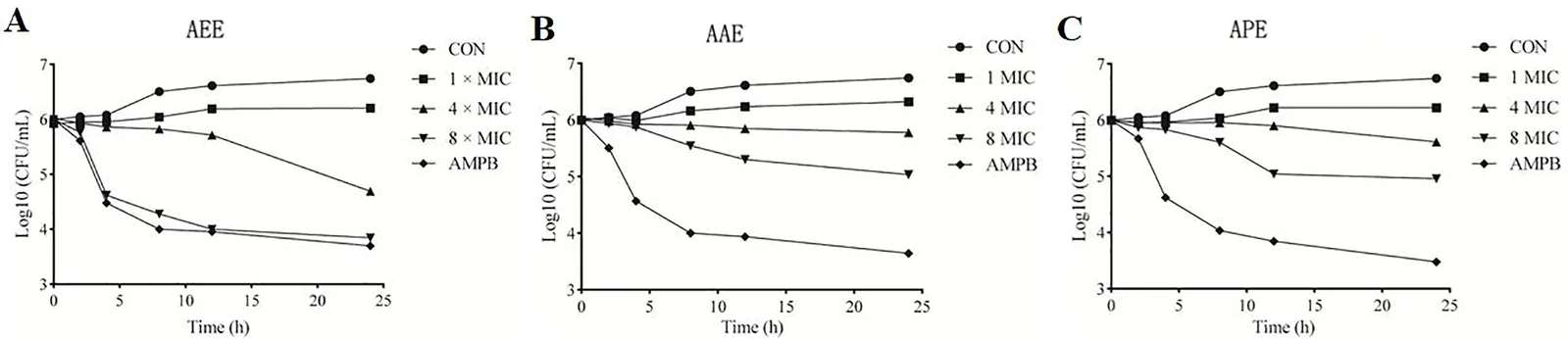
Time–kill curves of *Angelica dahurica–Ligusticum sinense* extracts against *Malassezia furfur.* **(A)** Ethanolic extract. (**B**) Aqueous extract. (**C**) Percolated extract.

The time-kill curve results demonstrated that the ethanol extract at a concentration of 8 × MIC maintained effective fungicidal activity against *M. furfur* throughout the 24-hour experimental period. The ethanolic extract at 1 × MIC and 4 × MIC concentrations showed certain inhibitory effects, although the effect at 1 × MIC was relatively weak. The water extract and percolation extract at 8 × MIC also exhibited some inhibitory activity, but no significant inhibition was observed at their respective 1 × MIC concentrations (Fig 1). The raw data underlying the time–kill curve analysis are provided in S1 Data.

### 3.3. Anti-biofilm activity of different extracts against *Malassezia furfur*

#### 3.3.1. Inhibition of biofilm formation.

**Fig 2:**
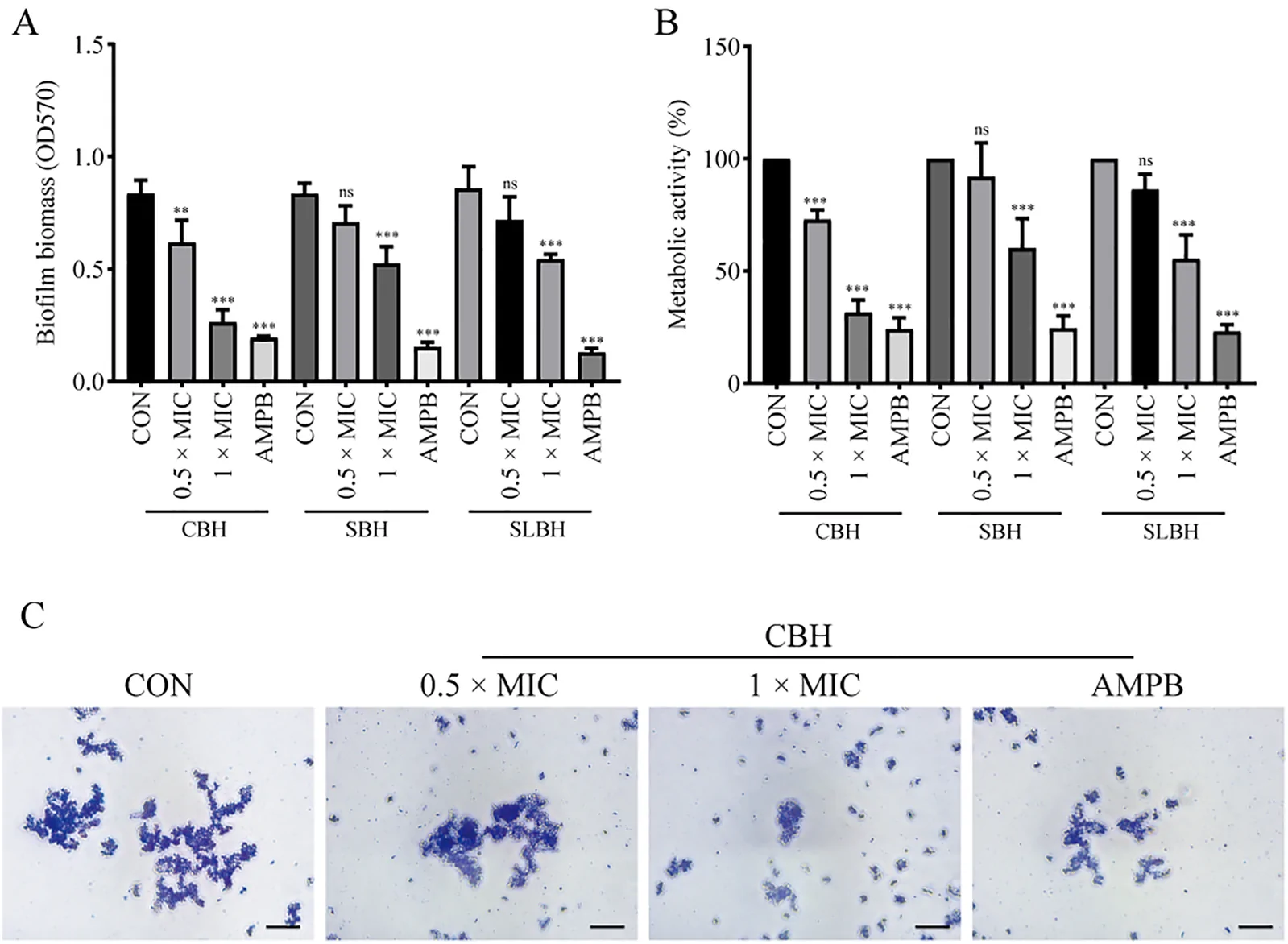
Inhibitory effects of *Angelica dahurica–Ligusticum sinense* extracts on *Malassezia furfur* biofilm formation. (**A**) Crystal-violet staining assay (CVA) evaluating the residual/biofilm-removal effect of different extracts. (**B**) Biofilm metabolic activity determined using the XTT reduction assay. (**C**) Representative light micrographs showing M. furfur biofilm formation in the presence of the ethanolic extract. AEE:ethanolic extract, AAE:aqueous extract, APE:percolation extract. ** p < 0.05,*** p < 0.01, n = 3.

Results from CVA and the XTT assay indicated that the ethanolic extract at 0.5 × MIC could inhibit biofilm formation, whereas the aqueous extract and percolation extract at the same concentration were ineffective. Under the tested conditions, AEE at 1 × MIC showed a biofilm-inhibitory effect comparable to that of amphotericin B in the biofilm formation assay. AAE and APE at 1 × MIC also inhibited biofilm formation. These findings suggest that AEE had the strongest inhibitory effect on biofilm formation. Microscopic observation further confirmed the significant inhibitory effect of AEE on *M. furfur* biofilm (Fig 2). The raw data underlying the CVA and XTT assays are provided in S1 Data.

#### 3.3.2. Reduction of mature biofilms.

**Fig 3:**
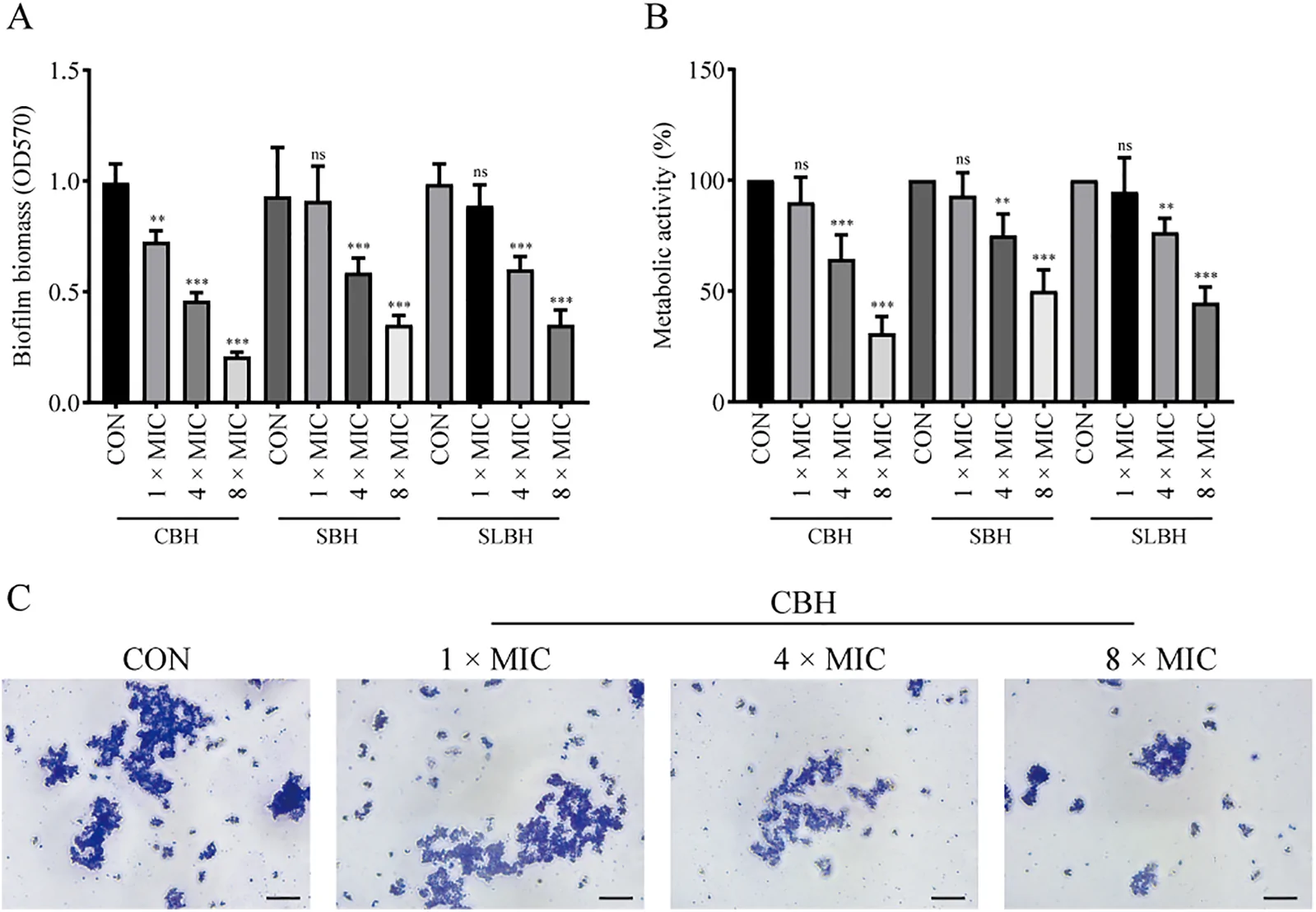
Effects of *Angelica dahurica–Ligusticum sinense* extracts on preformed *Malassezia furfur* biofilms. **(A)** Detection of the removal effect of different extracts on biofilm by the Crystal Violet Staining Assay (CVA). **(B)** Residual metabolic activity determined using the XTT reduction assay. **(C)** Representative light micrographs of preformed biofilms after treatment with the ethanolic extract. AEE:ethanolic extract, AAE:aqueous extract, APE:percolation extract. ** p < 0.05,*** p < 0.01.

Quantitative results from the CVA showed that the untreated control group had the highest biofilm biomass. Biofilm biomass gradually decreased with increasing extract concentration, with the most significant reduction observed in the group treated with the ethanolic extract at 8 × MIC. Results from the XTT metabolic activity analysis were consistent, showing the most pronounced decrease in metabolic activity in the group treated with the ethanolic extract at 8 × MIC. Microscopic observation revealed dense biofilms in the untreated group, minimal change was observed after treatment with the ethanol extract at 1 × MIC, whereas treatment with the ethanolic extract at 8 × MIC led to a significant reduction in biofilm biomass (Fig 3). The raw data underlying the CVA and XTT assays are provided in S1 Data.

### 3.4. Dermal toxicity of the ethanolic extract in rats

**Fig 4:**
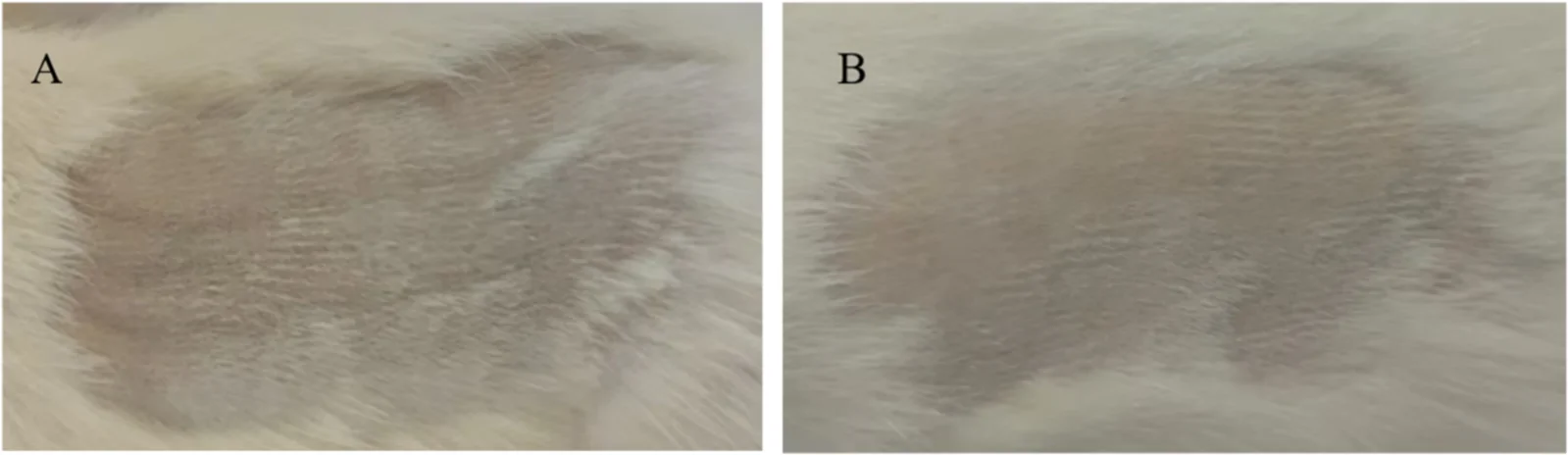
Dermal toxicity of the ethanol extract of *Angelica dahurica–Ligusticum sinense* in rats (A. Control group, B. Treated group).

During the entire observation period, no animals died and all remained in normal physiological condition. After removal of the preparation, the skin of both the treated group (8 × MIC) and the control group showed no erythema or oedema at 1, 24, 48 and 72 h (Fig 4). The irritation score for each group at every time-point was 0, giving a total irritation index of 0 (Table 3). These results indicate that the ethanol extract of *A.* *dahurica-L. sinense* at 8 × MIC showed no observable dermal irritation under the experimental conditions.

**Table 3:** Dermal irritation scoring in rats.

Group	Sex	Dose (MIC)	Total Score				Average Score			
			1 h	24 h	48 h	72 h	1 h	24 h	48 h	72 h
Control	♀		0	0	0	0	0	0	0	0
	♂		0	0	0	0	0	0	0	0
Treated	♀	8 MIC	0	0	0	0	0	0	0	0
	♂	8 MIC	0	0	0	0	0	0	0	0

## 4. Discussion

In this study, we systematically evaluated three extracts (ethanolic, aqueous, and cold-pressed) of the *A. dahurica-L. sinense* combination against *M. furfur* planktonic cells and biofilms. The ethanolic extract (AEE) exhibited the strongest antifungal activity, with a minimum inhibitory concentration (MIC) of 5 mg/mL, compared to 10 mg/mL for the other two extracts. UPLC analysis revealed that AEE contained markedly higher levels of five bioactive constituents—ferulic acid, bergapten, oxypeucedanin, imperatorin, and isoimperatorin—than the aqueous and cold-pressed extracts. These furanocoumarins and phenolic acids have previously been reported to possess antifungal properties [14–16]. Although the precise molecular mechanisms were not directly tested in our study, the superior performance of AEE is likely attributable to synergistic interactions among these lipophilic components, consistent with the multi-component, multi-target philosophy of traditional Chinese medicine [17].Time–kill kinetic curves further documented that AEE exerts concentration-dependent fungicidal activity, in sharp contrast to the merely fungistatic effect of certain conventional drugs [18] – a property that is crucial for rapid infection control and relapse prevention.

The most notable finding is that AEE effectively interfered with biofilm formation at 0.5 × MIC and eradicated pre-formed mature biofilms at 8 × MIC, as measured by biomass (crystal violet) and metabolic activity (XTT) assays. Microscopic examination confirmed a marked reduction in biofilm architecture. Biofilm-associated drug resistance is a major contributor to clinical treatment failure and recurrence in Malassezia-related skin diseases [5,19,20]. While conventional azoles often show limited penetration into biofilms [21,22], our results suggest that AEE may offer a potential strategy for addressing this recalcitrance. While our study did not directly investigate the underlying molecular mechanisms, coumarin compounds have previously been reported to interfere with initial adhesion [23,24], and ferulic acid has been shown to weaken biofilm structural integrity [25]. It is therefore plausible that the observed anti-biofilm effects of AEE involve these pathways, although direct head-to-head comparisons with clinical antifungals are needed before any claim of superiority can be made.

Importantly, no dermal irritation was observed in rats treated with 8 × MIC of AEE, indicating a favorable safety profile for topical application.This advantage makes it potentially more attractive than long-term use of synthetic antifungal agents (e.g., ketoconazole), which may cause skin dryness, irritation, and resistance [26].

Certainly, this study also has its limitations. First, all experiments were conducted in vitro; whether AEE exhibits comparable efficacy in vivo remains to be determined. Future studies should establish relevant animal models, such as a guinea pig model of *M. furfur* colonization or infection, to evaluate therapeutic potential and pharmacokinetic behavior [27]. Second, direct MFC comparisons between AEE and standard antifungal agents were not performed; therefore, further head-to-head studies are needed to more precisely evaluate its relative antifungal potency. Moreover, the underlying molecular mechanisms, e.g., the impact of AEE on biofilm-associated gene expression (e.g., adhesins, lipases), were not directly investigated and warrant further exploration [28].

## 5. Conclusion

In summary, this study evaluated the antifungal and antibiofilm activities of the *A. dahurica-L. sinense* AEE against *M. furfur*. AEE was enriched in coumarin and phenolic acid components and showed inhibitory activity against planktonic cells, reduced biofilm formation at relatively low concentrations, and decreased the biomass and metabolic activity of mature biofilms at higher concentrations. In addition, AEE caused no observable acute dermal irritation in rats under the experimental conditions tested. These findings provide preliminary experimental support for the further investigation of AEE as a potential natural topical agent for *M. furfur*-associated skin disorders, such as dandruff and seborrheic dermatitis.

## Supporting information

S1 Data Data. (XLSX)

S2 File Graphical abstract. (JPG)
