# Supplementary material for: Inhibitory effect and safety of Angelica dahurica–Ligusticum sinense extracts against Malassezia furfur and its biofilms
Source: PLoS One. 2026 Sep 15;21(9):e0357601. doi: 10.1371/journal.pone.0357601 (PMC13577356; doi:10.1371/journal.pone.0357601)
Supplement: Supplementary file 1 [file pone.0357601.s003.docx]

PONE-D-26-11512

Title:**Inhibitory Effect and Safety of *Angelica dahurica–Ligusticum sinense* Extracts Against *Malassezia furfur* and Its Biofilms**

Dear editor Rajesh P. Shastry,

We would like to thank you for your efforts in reviewing our manuscript titled "Inhibitory Effect and Safety of *Angelica dahurica–Ligusticum sinense* Extracts Against *Malassezia furfur* and Its Biofilms",and providing many helpful comments and suggestions, which will all prove invaluable in the revision and improvement of our paper, as well as in guiding our research in the future.

We have studied your comments point by point, revised the manuscript accordingly. The amendments are highlighted in red in the revised manuscript. All authors have approved the response letter and the revised version of the manuscript. To improve the precision of the title, we have changed the preposition “on” to “against”, as “against” more appropriately describes the antagonistic effect against Malassezia furfur and its biofilms. The revised title is provided in the manuscript.

We hope that the revised version of the manuscript is now acceptable for publication in PLOS One. If you have any queries, please do not hesitate to contact me.

Once again, we extend our heartfelt appreciation for your invaluable feedback. We eagerly anticipate your further guidance at your earliest convenience.

Yours sincerely,

Jinyan Zhang,

Dermatology Department

Fujian Provincial Geriatric Hospital

Email: [zjy_angel.220@163.com](mailto:zjy_angel.220@163.com)

Tel:+86-15205005836

**Response to Reviewer**

**For Editor**

**Point 1.** Please ensure that your manuscript meets PLOS ONE's style requirements, including those for file naming.

**Response 1:**

Thank you for this instruction. We have carefully revised the manuscript according to the PLOS ONE style requirements and checked the formatting, section organization, figure/table presentation, and file naming. The revised files have been prepared and named as required, including the marked-up revised manuscript, the clean manuscript, and the response letter.

**Point 2.** Please state what role the funders took in the study.  If the funders had no role, please state: ""The funders had no role in study design, data collection and analysis, decision to publish, or preparation of the manuscript."

**Response 2:**

Thank you for the reminder. We have revised the Funding statement to clarify the role of the funders. The revised statement reads: “The funders had no role in study design, data collection and analysis, decision to publish, or preparation of the manuscript.” This statement has also been included in the cover letter as required.( see Revised_without figures page 12, lines 342-343).

**Point 3.** Please complete your Competing Interests on the online submission form to state any Competing Interests.

**Response 3:**

Thank you for this instruction. We have revised the Competing Interests statement in the manuscript and cover letter as required. The statement now reads: “The authors have declared that no competing interests exist.” We will also ensure that the same statement is completed in the online submission form.( see Revised_without figures page 13, lines 354).

**Point 4&5.** In the online submission form, we indicated that the data would be provided upon request or made available upon acceptance. PLOS journals require all data underlying the findings to be freely available to researchers, either in a public repository, within the manuscript itself, or as Supporting Information.

**Response 4&5:**

Thank you for this important reminder. We have revised the Data Availability Statement to comply with the PLOS ONE data policy. The raw data underlying the findings of this study have been prepared and uploaded as Supporting Information. The revised statement now reads: “All relevant data are within the manuscript and its Supporting Information files. ”( see Revised_without figures page 13, lines 349-352 ).

**Point 6.** Please include captions for your Supporting Information files at the end of your manuscript, and update any in-text citations to match accordingly.

**Response 6:**

Thank you for this instruction. We have added captions for the Supporting Information files at the end of the manuscript and updated the corresponding in-text citations. The raw data underlying the quantitative analyses have been provided as Supporting Information and cited in the manuscript accordingly.( see Revised_without figures page 9, lines 243/255, page 10, lines 266, and page 13, lines 349-352 ).

**Point 7.** If the reviewer comments include a recommendation to cite specific previously published works, please review and evaluate these publications to determine whether they are relevant and should be cited. There is no requirement to cite these works unless the editor has indicated otherwise.

**Response 7:**
Thank you for this reminder. The reviewers did not recommend any specific previously published works for citation. We have nevertheless carefully reviewed the reference list and revised the manuscript where necessary to ensure that the cited literature is relevant and appropriate.

**Reviewer #1**

**Point 1.** Some sections would benefit from language polishing to improve clarity and readability.

**Response 1:**

Thank you for this helpful suggestion. We have carefully polished the language throughout the manuscript to improve clarity, readability, and grammatical accuracy. In particular, we revised the Abstract, Introduction, Results, Discussion, and Conclusion sections to make the scientific expression more concise and precise. We also standardized terminology and corrected typographical and formatting issues throughout the manuscript.( see Revised_without figures )

**Point 2.** The number of experimental replicates and details of statistical analyses should be described more clearly. **Response 2:**

Thank you for this helpful suggestion. We have clarified the number of experimental replicates in the Methods section and the corresponding figure/table legends. Specifically, we now state that the key experiments were performed with three independent replicates. We have also revised the Statistical analysis section to describe the data presentation and statistical methods more clearly.( see Revised_without figures page 8, lines 211-212 ).

**Point 3.** It would be useful to compare the antifungal activity (e.g., MIC/MFC values) with those of standard antifungal agents or previously reported studies.
**Response 3:**

Thank you for this valuable comment. We agree that comparison with standard antifungal agents is important for interpreting the antifungal activity of AEE. In this study, amphotericin B was included as a positive control in the time–kill curve assay and the biofilm inhibition assay. We have revised the Results and Discussion sections to make this comparison clearer. The revised manuscript now states that AEE showed marked inhibitory activity in the time–kill assay and that AEE at 1× MIC exhibited biofilm inhibition comparable to amphotericin B under the tested conditions. At the same time, we have tempered the interpretation of these findings, because direct MFC comparisons with amphotericin B or other standard antifungal agents were not performed in this study. We have also added this point to the limitations and suggested that future studies should include head-to-head MIC/MFC comparisons with clinically used antifungal drugs.( see Revised_without figures page 11, lines 315-317 )

**Point 4.** The limitations of in vitro experiments should be acknowledged, with suggestions for future in vivo validation.
**Response4:**

Thank you for this important comment. We agree that the in vitro nature of the current study should be clearly acknowledged. We have expanded the limitations section to acknowledge that the antifungal and antibiofilm activities were evaluated mainly in vitro and that in vivo infection models are needed. ( see Revised_without figures page 11, lines 311-314 )

**Reviewer #2**

**Point 1.** Mechanistic claims should be tempered. The Discussion attributes antifungal and antibiofilm effects to specific mechanisms, such as disruption of adhesion, interference with the extracellular matrix, and gene regulation, but these mechanisms are not directly tested. These statements should be reframed as hypotheses rather than conclusions, or clearly identified as speculative.

**Response1：**

Thank you for this important comment. We agree that the molecular mechanisms underlying the antifungal and antibiofilm effects of AEE were not directly investigated in the present study. Therefore, we have revised the Discussion to avoid definitive mechanistic conclusions. Statements related to adhesion, extracellular matrix formation, and biofilm-associated gene regulation have been reframed as hypotheses and clearly identified as areas for future investigation. (see Revised_without figures page 11, lines 300-302/317-320 )

**Point 2.** Biofilm conclusions should align strictly with the data.
The CV, XTT, and microscopy data convincingly demonstrate biofilm inhibition and eradication. However, conclusions implying molecular or structural targeting go beyond what these assays support. The authors should clarify that the findings are based on biomass and metabolic activity measurements.

**Response 2：**

Thank you for this important comment. We agree that the biofilm-related conclusions should be strictly aligned with the experimental data. We have revised the Results, Discussion, and Conclusion to ensure that the biofilm conclusions are strictly aligned with the data. We now state that AEE reduced biofilm biomass and metabolic activity, as measured by CVSA and XTT assays, respectively, with supporting microscopic observations. Claims implying specific molecular or structural targeting have been removed or rephrased as hypotheses. (see Revised_without figures page 11, lines 293-300 )

**Point 3.** Claims regarding clinical advantage need moderation.
While the ethanolic extract shows strong antibiofilm activity, comparisons with clinical azoles and claims of overcoming "drug resistance" should be toned down, as resistant clinical isolates or head‑to‑head efficacy studies were not conducted.

**Response 3：**

Thank you for this important comment. We agree that the clinical implications of our findings should be interpreted cautiously. In the revised manuscript, we have toned down statements regarding the clinical advantage of the ethanolic extract (AEE) and removed claims suggesting that AEE can overcome antifungal drug resistance. We have also avoided direct comparisons with clinical azoles, as resistant clinical isolates and head-to-head efficacy studies with standard antifungal agents were not included in the present study. The revised manuscript now presents AEE as a potential candidate for further investigation rather than as a clinically superior alternative. (see Revised_without figures page 11, lines 298-300 )

**Point 4.** Discussion is overly long and repetitive.

**Response 4：**

Thank you for this valuable suggestion. We have substantially condensed the Discussion section and removed repetitive background information that had already been addressed in the Introduction. The revised Discussion now focuses on the three key findings highlighted by the reviewer: first, ethanol extraction yielded the most active preparation; second, AEE reduced both biofilm formation and mature biofilm biomass/metabolic activity; and third, AEE showed good dermal tolerability in the acute rat skin irritation model. We have also revised the Discussion to avoid overinterpretation of mechanistic and clinical implications.(see Revised_without figures page 10-11 )

**Point 5.** Species names throughout the manuscript need to be formatted correctly as well as in the 'Graphical Abstract Image' (Example: *Malassezia furfur* is not italicized).Reduce repetition of background material already covered in the Introduction.Ensure consistent terminology (e.g., use "ethanolic extract (AEE)" throughout after the first definition).Clarify the number of biological replicates for key assays.

**Response 5：**

Thank you for these helpful suggestions. We have carefully revised the manuscript accordingly. First, we checked and corrected the formatting of all species names throughout the manuscript and in the Graphical Abstract Image, including *Malassezia furfur, Angelica dahurica, and Ligusticum sinense*. Second, we removed repetitive background information from the Discussion section and focused the revised Discussion on the main findings of the study. Third, we standardized the terminology throughout the manuscript; after the first definition, we consistently use “ethanolic extract (AEE),” “aqueous extract (AAE),” and “percolation extract (APE).” Finally, we clarified the number of biological replicates for the key assays in the Methods section and the relevant figure/table legends. The revised manuscript now states that the key experiments were performed with three independent replicates. (see Revised_without figures)
