# Supplementary material for: Inhibitory effect and safety of Angelica dahurica–Ligusticum sinense extracts against Malassezia furfur and its biofilms
Source: PLoS One. 2026 Sep 15;21(9):e0357601. doi: 10.1371/journal.pone.0357601 (PMC13577356; doi:10.1371/journal.pone.0357601)
Supplement: Supplementary file 2 [file pone.0357601.s004.docx]

**Response to Reviewer**

**Reviewer #3**

**Point 1. It has not been specified which strain of Malassezia furfur was used; its origin is unknown, nor is it clear whether it was typed.**

**Response 1:**

Thank you for identifying this omission. We have added a new subsection entitled “Fungal strain” to the Materials and Methods section. The M. furfur isolate used in this study (MF-FZ12) was obtained from the archived fungal collection of Dermatology hospital of Fuzhou, Fuzhou First General Hospital Affiliated with Fujian Medical University, Fuzhou, China. ( Page 6, Line 158-166)

**Point 2: Figures 2 and 3 appear to be identical, although their captions describe different experiments. The Materials and Methods section describes only biofilm inhibition. If preformed biofilm is to be studied, further experiments must be conducted.**

**Response 2:**

We apologize for the confusion caused by the incorrect Figure 2 legend and the omission of the preformed-biofilm protocol from the Materials and Methods section. Figures 2 and 3 represent two distinct experimental designs. In the biofilm-formation inhibition experiment shown in Figure 2, the extracts were added at the time of inoculation and remained present during biofilm formation. In the preformed-biofilm experiment shown in Figure 3, biofilms were first established in the absence of the extracts, washed, and subsequently exposed to the extracts.

We have corrected the Figure 2 title and legend so that they consistently describe inhibition of biofilm formation. We have also added a detailed subsection describing the establishment and treatment of preformed biofilms, including the sequence of incubation, washing, extract treatment, controls, and CVA/XTT measurements.(see Page 8, Line 206-216) .The Figure 3 legend has been revised to clearly identify it as the experiment performed on preformed biofilms. In addition, to avoid overstatement, we have replaced “eradication” with “reduction of preformed-biofilm biomass and metabolic activity” where appropriate. The two figure files have been checked and re-uploaded separately to ensure that the correct images are displayed.

**Point 3: The abbreviation for the crystal violet assay is simply CVA.**

**Response 3:**

We agree. “Crystal violet staining assay (CVSA)” has been changed to “crystal violet assay (CVA)” at its first occurrence, and the abbreviation “CVA” is now used consistently throughout the manuscript, including the Methods, Results, and figure legends.
