# Supplementary material for: Inhibitory effect and safety of Angelica dahurica–Ligusticum sinense extracts against Malassezia furfur and its biofilms
Source: PLoS One. 2026 Sep 15;21(9):e0357601. doi: 10.1371/journal.pone.0357601 (PMC13577356; doi:10.1371/journal.pone.0357601)
Supplement: Peer review history

# Peer review: Decision Letter 0

**DOI:** 10.1371/journal.pone.0357601.r001

**Authors:** Rajesh P. Shastry

27 Apr 2026

Dear Dr. Zhang,

Thank you for submitting your manuscript to PLOS ONE. After careful consideration, we feel that it has merit but does not fully meet PLOS ONE’s publication criteria as it currently stands. Therefore, we invite you to submit a revised version of the manuscript that addresses the points raised during the review process.

Please submit your revised manuscript by Jun 11 2026 11:59PM. If you will need more time than this to complete your revisions, please reply to this message or contact the journal office at plosone@plos.org. When you're ready to submit your revision, log on to https://www.editorialmanager.com/pone/ and select the 'Submissions Needing Revision' folder to locate your manuscript file.

- A letter that responds to each point raised by the academic editor and reviewer(s). You should upload this letter as a separate file labeled 'Response to Reviewers'.
- A marked-up copy of your manuscript that highlights changes made to the original version. You should upload this as a separate file labeled 'Revised Manuscript with Track Changes'.
- An unmarked version of your revised paper without tracked changes. You should upload this as a separate file labeled 'Manuscript'.

If you would like to make changes to your financial disclosure, please include your updated statement in your cover letter. Guidelines for resubmitting your figure files are available below the reviewer comments at the end of this letter.

If applicable, we recommend that you deposit your laboratory protocols in protocols.io to enhance the reproducibility of your results. Protocols.io assigns your protocol its own identifier (DOI) so that it can be cited independently in the future. For instructions see: https://journals.plos.org/plosone/s/submission-guidelines#loc-laboratory-protocols. Additionally, PLOS ONE offers an option for publishing peer-reviewed Lab Protocol articles, which describe protocols hosted on protocols.io. Read more information on sharing protocols at https://plos.org/protocols?utm_medium=editorial-email&utm_source=authorletters&utm_campaign=protocols.

As the corresponding author, your ORCID iD is verified in the submission system and will appear in the published article. PLOS supports the use of ORCID, and we encourage all coauthors to register for an ORCID iD and use it as well. Please encourage your coauthors to verify their ORCID iD within the submission system before final acceptance, as unverified ORCID iDs will not appear in the published article. *Only*  the individual author can complete the verification step; PLOS staff *cannot*  verify ORCID iDs on behalf of authors.

We look forward to receiving your revised manuscript.

Kind regards,

Rajesh P. Shastry, Ph.D

Academic Editor

PLOS One

Journal Requirements:

When submitting your revision, we need you to address these additional requirements.

1. Please ensure that your manuscript meets PLOS ONE's style requirements, including those for file naming. The PLOS ONE style templates can be found at

https://journals.plos.org/plosone/s/file?id=wjVg/PLOSOne_formatting_sample_main_body.pdf and

https://journals.plos.org/plosone/s/file?id=ba62/PLOSOne_formatting_sample_title_authors_affiliations.pdf

2. Thank you for stating the following financial disclosure: [copy in funding statement].

Please state what role the funders took in the study.  If the funders had no role, please state: ""The funders had no role in study design, data collection and analysis, decision to publish, or preparation of the manuscript.""

If this statement is not correct you must amend it as needed.

Please include this amended Role of Funder statement in your cover letter; we will change the online submission form on your behalf.

3. Thank you for stating the following in your Competing Interests section: [NO authors have competing interests].

Please complete your Competing Interests on the online submission form to state any Competing Interests. If you have no competing interests, please state ""The authors have declared that no competing interests exist."", as detailed online in our guide for authors at http://journals.plos.org/plosone/s/submit-now

This information should be included in your cover letter; we will change the online submission form on your behalf.

4. In the online submission form, you indicated that [We will provide the data necessary to replicate the reported findings to any interested party upon request.].

All PLOS journals now require all data underlying the findings described in their manuscript to be freely available to other researchers, either 1. In a public repository, 2. Within the manuscript itself, or 3. Uploaded as supplementary information.

This policy applies to all data except where public deposition would breach compliance with the protocol approved by your research ethics board. If your data cannot be made publicly available for ethical or legal reasons (e.g., public availability would compromise patient privacy), please explain your reasons on resubmission and your exemption request will be escalated for approval.

5. When completing the data availability statement of the submission form, you indicated that you will make your data available on acceptance. We strongly recommend all authors decide on a data sharing plan before acceptance, as the process can be lengthy and hold up publication timelines. Please note that, though access restrictions are acceptable now, your entire data will need to be made freely accessible if your manuscript is accepted for publication. This policy applies to all data except where public deposition would breach compliance with the protocol approved by your research ethics board. If you are unable to adhere to our open data policy, please kindly revise your statement to explain your reasoning and we will seek the editor's input on an exemption. Please be assured that, once you have provided your new statement, the assessment of your exemption will not hold up the peer review process.

6. Please include captions for your Supporting Information files at the end of your manuscript, and update any in-text citations to match accordingly. Please see our Supporting Information guidelines for more information: http://journals.plos.org/plosone/s/supporting-information.

7. If the reviewer comments include a recommendation to cite specific previously published works, please review and evaluate these publications to determine whether they are relevant and should be cited. There is no requirement to cite these works unless the editor has indicated otherwise.

[Note: HTML markup is below. Please do not edit.]

Reviewers' comments:

Reviewer's Responses to Questions

**Comments to the Author**

1. Is the manuscript technically sound, and do the data support the conclusions?

Reviewer #1: Yes

Reviewer #2: Yes

**********

2. Has the statistical analysis been performed appropriately and rigorously?

Reviewer #1: Yes

Reviewer #2: Yes

**********

3. Have the authors made all data underlying the findings in their manuscript fully available?

The PLOS Data policy

Reviewer #1: Yes

Reviewer #2: Yes

**********

4. Is the manuscript presented in an intelligible fashion and written in standard English?

Reviewer #1: Yes

Reviewer #2: Yes

**********

Reviewer #1: I found this manuscript to be interesting, well-structured, and scientifically sound. The authors investigate the antifungal and anti-biofilm activity of Angelica dahurica and Ligusticum sinense extracts against Malassezia furfur, a clinically relevant pathogen associated with dermatological conditions.

The topic is timely and relevant, particularly in the context of increasing antifungal resistance and the growing interest in plant-derived therapeutic alternatives. Overall, the study is well designed, and the experimental approach is appropriate to address the research questions. The results are clearly presented and easy to follow, and the conclusions are generally well supported by the data.

One of the main strengths of this study lies in the comprehensive evaluation of antifungal activity using multiple complementary experimental approaches. The comparison between different extraction methods provides valuable insight into how extraction strategy influences bioactivity.

The identification of active compounds using HPLC significantly strengthens the scientific value and reproducibility of the study.

I also appreciate the inclusion of biofilm inhibition and eradication assays, which are highly relevant from a clinical perspective since biofilms are a major factor in antifungal resistance and treatment failure.

Additionally, the inclusion of a preliminary safety assessment, although limited, adds further value to the manuscript.

The manuscript is generally of good quality; however, a few minor improvements are suggested:

Some sections would benefit from language polishing to improve clarity and readability.

The number of experimental replicates and details of statistical analyses should be described more clearly.

It would be useful to compare the antifungal activity (e.g., MIC/MFC values) with those of standard antifungal agents or previously reported studies.

The limitations of in vitro experiments should be acknowledged, with suggestions for future in vivo validation.

The data availability statement should be clarified to ensure full compliance with journal requirements.

In my opinion, this is a solid and well-executed study that provides useful and reliable data in the field of natural antifungal agents. The manuscript contributes valuable insights into plant-based antifungal strategies.

Reviewer #2: Recommendation: Revise

This manuscript presents a thorough in vitro evaluation of Angelica dahurica–Ligusticum sinense extracts against Malassezia furfur, including planktonic growth, biofilm inhibition and eradication, chemical profiling, and preliminary dermal safety testing. The topic is relevant and timely given Malassezia biofilms' role in treatment failure and recurrence. Overall, the experiments are well executed, but several aspects of interpretation and presentation require revision.

Major comments:

Mechanistic claims should be tempered.

The Discussion attributes antifungal and antibiofilm effects to specific mechanisms (e.g., disruption of adhesion, interference with the extracellular matrix, gene regulation), but these mechanisms are not directly tested. These statements should be reframed as hypotheses rather than conclusions, or clearly identified as speculative.

Biofilm conclusions should align strictly with the data.

The CV, XTT, and microscopy data convincingly demonstrate biofilm inhibition and eradication. However, conclusions implying molecular or structural targeting go beyond what these assays support. The authors should clarify that the findings are based on biomass and metabolic activity measurements.

Claims regarding clinical advantage need moderation.

While the ethanolic extract shows strong antibiofilm activity, comparisons with clinical azoles and claims of overcoming "drug resistance" should be toned down, as resistant clinical isolates or head‑to‑head efficacy studies were not conducted.

Discussion is overly long and repetitive.

The Discussion would benefit from substantial condensation, focusing on three key findings: (i) ethanol extraction yields the most active preparation, (ii) the extract is effective against both forming and mature biofilms, and (iii) the extract shows good dermal tolerability.

Minor comments:

Species names throughout the manuscript need to be formatted correctly as well as in the 'Graphical Abstract Image' (Example: Malassezia furfur is not italicized).

Reduce repetition of background material already covered in the Introduction.

Ensure consistent terminology (e.g., use "ethanolic extract (AEE)" throughout after the first definition).

Clarify the number of biological replicates for key assays.

Overall assessment:

The study provides solid in vitro evidence supporting the antifungal and antibiofilm activities of a traditional herbal combination. With clearer framing of limitations and more cautious interpretation of mechanisms and clinical relevance, the manuscript would be suitable for publication.

Willingness to review a revision: Yes.

**********

what does this mean?). If published, this will include your full peer review and any attached files.

If you choose “no”, your identity will remain anonymous but your review may still be made public.

**Do you want your identity to be public for this peer review?**  For information about this choice, including consent withdrawal, please see our Privacy Policy

Reviewer #1: No

Reviewer #2: No

**********

[NOTE: If reviewer comments were submitted as an attachment file, they will be attached to this email and accessible via the submission site. Please log into your account, locate the manuscript record, and check for the action link "View Attachments". If this link does not appear, there are no attachment files.]

To ensure your figures meet our technical requirements, please review our figure guidelines: https://journals.plos.org/plosone/s/figures

You may also use PLOS’s free figure tool, NAAS, to help you prepare publication quality figures: https://journals.plos.org/plosone/s/figures#loc-tools-for-figure-preparation.

NAAS will assess whether your figures meet our technical requirements by comparing each figure against our figure specifications.

# Author comment: Author response to Decision Letter 1

**DOI:** 10.1371/journal.pone.0357601.r002

25 May 2026

Dear Editor,

Thank you for the opportunity to revise our manuscript. We have carefully addressed all comments from the Academic Editor and reviewers. The revised manuscript includes language polishing, clarification of experimental replicates and statistical analyses, moderation of mechanistic and clinical claims, condensation of the Discussion, revision of biofilm-related conclusions, and standardization of species-name formatting and terminology. We have also updated the Funding, Competing Interests, and Data Availability statements, and uploaded the raw data as Supporting Information. A detailed point-by-point response, a marked-up manuscript, and a clean manuscript have been uploaded as required.

Sincerely,

Huan Li

Attachment Submitted filename: Response to Reviewers.docx

# Peer review: Decision Letter 1

**DOI:** 10.1371/journal.pone.0357601.r003

**Authors:** Rajesh P. Shastry

22 Jul 2026

Dear Dr. Li,

Thank you for submitting your manuscript to PLOS ONE. After careful consideration, we feel that it has merit but does not fully meet PLOS ONE’s publication criteria as it currently stands. Therefore, we invite you to submit a revised version of the manuscript that addresses the points raised during the review process.

Please submit your revised manuscript by Sep 05 2026 11:59PM. If you will need more time than this to complete your revisions, please reply to this message or contact the journal office at plosone@plos.org. When you're ready to submit your revision, log on to https://www.editorialmanager.com/pone/ and select the 'Submissions Needing Revision' folder to locate your manuscript file.

- A letter that responds to each point raised by the academic editor and reviewer(s). You should upload this letter as a separate file labeled 'Response to Reviewers'.
- A marked-up copy of your manuscript that highlights changes made to the original version. You should upload this as a separate file labeled 'Revised Manuscript with Track Changes'.
- An unmarked version of your revised paper without tracked changes. You should upload this as a separate file labeled 'Manuscript'.

If you would like to make changes to your financial disclosure, please include your updated statement in your cover letter. Guidelines for resubmitting your figure files are available below the reviewer comments at the end of this letter.

If applicable, we recommend that you deposit your laboratory protocols in protocols.io to enhance the reproducibility of your results. Protocols.io assigns your protocol its own identifier (DOI) so that it can be cited independently in the future. For instructions see: https://journals.plos.org/plosone/s/submission-guidelines#loc-laboratory-protocols. Additionally, PLOS ONE offers an option for publishing peer-reviewed Lab Protocol articles, which describe protocols hosted on protocols.io. Read more information on sharing protocols at https://plos.org/protocols?utm_medium=editorial-email&utm_source=authorletters&utm_campaign=protocols.

As the corresponding author, your ORCID iD is verified in the submission system and will appear in the published article. PLOS supports the use of ORCID, and we encourage all coauthors to register for an ORCID iD and use it as well. Please encourage your coauthors to verify their ORCID iD within the submission system before final acceptance, as unverified ORCID iDs will not appear in the published article. *Only*  the individual author can complete the verification step; PLOS staff *cannot*  verify ORCID iDs on behalf of authors.

We look forward to receiving your revised manuscript.

Kind regards,

Rajesh P. Shastry, Ph.D

Academic Editor

PLOS One

Journal Requirements:

If the reviewer comments include a recommendation to cite specific previously published works, please review and evaluate these publications to determine whether they are relevant and should be cited. There is no requirement to cite these works unless the editor has indicated otherwise.

Please review your reference list to ensure that it is complete and correct. If you have cited papers that have been retracted, please include the rationale for doing so in the manuscript text, or remove these references and replace them with relevant current references. Any changes to the reference list should be mentioned in the rebuttal letter that accompanies your revised manuscript. If you need to cite a retracted article, indicate the article’s retracted status in the References list and also include a citation and full reference for the retraction notice.

[Note: HTML markup is below. Please do not edit.]

Reviewers' comments:

Reviewer's Responses to Questions

**Comments to the Author**

Reviewer #3: All comments have been addressed

**********

2. Is the manuscript technically sound, and do the data support the conclusions?

Reviewer #3: Partly

**********

3. Has the statistical analysis been performed appropriately and rigorously?

Reviewer #3: N/A

**********

4. Have the authors made all data underlying the findings in their manuscript fully available?

The PLOS Data policy

Reviewer #3: Yes

**********

5. Is the manuscript presented in an intelligible fashion and written in standard English?

Reviewer #3: Yes

**********

Reviewer #3: The manuscript still requires further revision as some information is missing.

1) It has not been specified which strain of Malassezia furfur was used; its origin is unknown, nor is it clear whether it was typed.

2) The text includes two figures, Figures 2 and 3, which appear to be identical; however, the captions describe Figure 2 as the inhibition of biofilm in the presence of the extract and Figure 3 as the eradication of biofilm.

The ‘Materials and Methods’ section describes only biofilm inhibition; nothing else.

If you wish to study preformed biofilm, further experiments must be conducted.

3) The abbreviation for the crystal violet assay is simply (CVA)

**********

what does this mean?). If published, this will include your full peer review and any attached files.

If you choose “no”, your identity will remain anonymous but your review may still be made public.

**Do you want your identity to be public for this peer review?**  For information about this choice, including consent withdrawal, please see our Privacy Policy

Reviewer #3: No

**********

[NOTE: If reviewer comments were submitted as an attachment file, they will be attached to this email and accessible via the submission site. Please log into your account, locate the manuscript record, and check for the action link "View Attachments". If this link does not appear, there are no attachment files.]

To ensure your figures meet our technical requirements, please review our figure guidelines: https://journals.plos.org/plosone/s/figures

You may also use PLOS’s free figure tool, NAAS, to help you prepare publication quality figures: https://journals.plos.org/plosone/s/figures#loc-tools-for-figure-preparation.

NAAS will assess whether your figures meet our technical requirements by comparing each figure against our figure specifications.

# Author comment: Author response to Decision Letter 2

**DOI:** 10.1371/journal.pone.0357601.r004

14 Aug 2026

Point 1. It has not been specified which strain of Malassezia furfur was used; its origin is unknown, nor is it clear whether it was typed.

Response 1:

Thank you for identifying this omission. We have added a new subsection entitled “Fungal strain” to the Materials and Methods section. The M. furfur isolate used in this study (MF-FZ12) was obtained from the archived fungal collection of Dermatology hospital of Fuzhou, Fuzhou First General Hospital Affiliated with Fujian Medical University, Fuzhou, China. ( Page 6, Line 158-166)

Point 2: Figures 2 and 3 appear to be identical, although their captions describe different experiments. The Materials and Methods section describes only biofilm inhibition. If preformed biofilm is to be studied, further experiments must be conducted.

Response 2:

We apologize for the confusion caused by the incorrect Figure 2 legend and the omission of the preformed-biofilm protocol from the Materials and Methods section. Figures 2 and 3 represent two distinct experimental designs. In the biofilm-formation inhibition experiment shown in Figure 2, the extracts were added at the time of inoculation and remained present during biofilm formation. In the preformed-biofilm experiment shown in Figure 3, biofilms were first established in the absence of the extracts, washed, and subsequently exposed to the extracts.

We have corrected the Figure 2 title and legend so that they consistently describe inhibition of biofilm formation. We have also added a detailed subsection describing the establishment and treatment of preformed biofilms, including the sequence of incubation, washing, extract treatment, controls, and CVA/XTT measurements.(see Page 8, Line 206-216) .The Figure 3 legend has been revised to clearly identify it as the experiment performed on preformed biofilms. In addition, to avoid overstatement, we have replaced “eradication” with “reduction of preformed-biofilm biomass and metabolic activity” where appropriate. The two figure files have been checked and re-uploaded separately to ensure that the correct images are displayed.

Point 3: The abbreviation for the crystal violet assay is simply CVA.

Response 3:

We agree. “Crystal violet staining assay (CVSA)” has been changed to “crystal violet assay (CVA)” at its first occurrence, and the abbreviation “CVA” is now used consistently throughout the manuscript, including the Methods, Results, and figure legends.

Attachment Submitted filename: Response to Reviewer.docx

# Editor report: Decision Letter 2

**DOI:** 10.1371/journal.pone.0357601.r005

**Authors:** Rajesh P. Shastry

19 Aug 2026

Inhibitory effect and safety of Angelica dahurica–Ligusticum sinense  extracts against Malassezia furfur  and its biofilms

PONE-D-26-11512R2

Dear Dr. Li,

We’re pleased to inform you that your manuscript has been judged scientifically suitable for publication and will be formally accepted for publication once it meets all outstanding technical requirements.

Within one week, you’ll receive an e-mail detailing the required amendments. When these have been addressed, you’ll receive a formal acceptance letter and your manuscript will be scheduled for publication.

An invoice will be generated when your article is formally accepted. Please note, if your institution has a publishing partnership with PLOS and your article meets the relevant criteria, all or part of your publication costs will be covered. Please make sure your user information is up-to-date by logging into Editorial Manager at Editorial Manager® and clicking the ‘Update My Information' link at the top of the page. For questions related to billing, please contact billing support.

If your institution or institutions have a press office, please notify them about your upcoming paper to help maximize its impact. If they’ll be preparing press materials, please inform our press team as soon as possible -- no later than 48 hours after receiving the formal acceptance. Your manuscript will remain under strict press embargo until 2 pm Eastern Time on the date of publication. For more information, please contact onepress@plos.org.

Kind regards,

Rajesh P. Shastry, Ph.D

Academic Editor

PLOS One

Additional Editor Comments (optional):

Reviewers' comments:

# Editor report: Acceptance letter

**DOI:** 10.1371/journal.pone.0357601.r006

**Authors:** Rajesh P. Shastry

PONE-D-26-11512R2

PLOS One

Dear Dr. Li,

I'm pleased to inform you that your manuscript has been deemed suitable for publication in PLOS One. Congratulations! Your manuscript is now being handed over to our production team.

At this stage, our production department will prepare your paper for publication. This includes ensuring the following:

* All references, tables, and figures are properly cited

* All relevant supporting information is included in the manuscript submission,

* There are no issues that prevent the paper from being properly typeset

You will receive further instructions from the production team, including instructions on how to review your proof when it is ready. Please keep in mind that we are working through a large volume of accepted articles, so please give us a few days to review your paper and let you know the next and final steps.

Lastly, if your institution or institutions have a press office, please let them know about your upcoming paper now to help maximize its impact. If they'll be preparing press materials, please inform our press team within the next 48 hours. Your manuscript will remain under strict press embargo until 2 pm Eastern Time on the date of publication. For more information, please contact onepress@plos.org.

You will receive an invoice from PLOS for your publication fee after your manuscript has reached the completed accept phase. If you receive an email requesting payment before acceptance or for any other service, this may be a phishing scheme. Learn how to identify phishing emails and protect your accounts at https://explore.plos.org/phishing.

If we can help with anything else, please email us at customercare@plos.org.

Thank you for submitting your work to PLOS One and supporting open access.

Kind regards,

PLOS One Editorial Office Staff

on behalf of

Dr. Rajesh P. Shastry

Academic Editor

PLOS One
